# Efficacy and safety of early ultrafiltration in patients with acute decompensated heart failure with volume overload: a prospective, randomized, controlled clinical trial

**DOI:** 10.1186/s12872-020-01733-5

**Published:** 2020-10-14

**Authors:** Jingyi Hu, Qianli Wan, Yue Zhang, Jun Zhou, Miaomiao Li, Li Jiang, Fang Yuan

**Affiliations:** 1 Department of Critical Care Medicine (Specialty of Heart Failure), Tongren Hospital, Shanghai Jiaotong University School of Medicine, No. 1111 Xianxia Road, Shanghai, 200336 China; 2 Department of Cardiology, Tongren Hospital, Shanghai Jiaotong University School of Medicine, No. 1111 Xianxia Road, Shanghai, 200336 China

**Keywords:** Early ultrafiltration, Heart failure, Fluid overload, Loop diuretics, Sequential therapy

## Abstract

**Background:**

Ultrafiltration decreases total body water and improves the alveolar to arterial oxygen gradient. The aims of the study were to investigate the efficacy and safety of early ultrafiltration in acute decompensated heart failure (ADHF) patients.

**Methods:**

100 patients with ADHF within 24 h of admission were randomly assigned into early ultrafiltration (n = 40) or torasemide plus tolvaptan (n = 60) groups. The primary outcomes were weight loss and an increase in urine output on days 4 and 8 of treatment.

**Results:**

Patients who received early ultrafiltration for 3 days achieved a greater weight loss (kg) (− 2.94 ± 3.76 vs − 0.64 ± 0.91, *P* < 0.001) and urine increase (mL) (198.00 ± 170.70 vs 61.77 ± 4.67, *P* < 0.001) than the torasemide plus tolvaptan group on day 4. From days 4 to 7, patients in the early ultrafiltration group received sequential therapy of torasemide and tolvaptan. Better control of volume was reflected in a greater weight loss (− 3.72 ± 3.81 vs − 1.34 ± 1.32, *P* < 0.001) and urine increase (373.80 ± 120.90 vs 79.5 ± 52.35, *P* < 0.001), greater reduction of B-type natriuretic peptide (BNP) (pg/mL) (− 1144 ± 1435 vs − 654.02 ± 889.65, *P* = 0.037), NYHA (New York Heart Association) functional class (− 1.45 ± 0.50 vs − 1.17 ± 0.62, *P* = 0.018), jugular venous pulse (JVP) score (points) (− 1.9 ± 1.13 vs − 0.78 ± 0.69, *P* < 0.001), inferior vena cava (IVC) diameter (mm) (− 15.35 ± 11.03 vs − 4.98 ± 6.00, *P* < 0.001) and an increase in the dyspnea score (points) (4.08 ± 3.44 vs 2.77 ± 2.03, *P* = 0.035) in the early ultrafiltration group on day 8. No significant differences were found in the readmission and mortality rates in the 2 patient groups at the 1-month and 3-month follow-ups. Both groups had a similar stable renal profile.

**Conclusion:**

Early ultrafiltration is superior to diuretics for volume overload treatment initiation of ADHF patients.

*Trial registration* Chinese Clinical Trial Registry, ChiCTR2000030696, Registered 10 March 2020—Retrospectively registered, https://www.chictr.org.cn/showproj.aspx?proj=29099.

## Background

Acute heart failure (AHF) arises from a variety of causes. Clinical symptoms of heart failure (HF) occur rapidly or exacerbate, accompanied by elevated plasma natriuretic peptide levels, which are often life threatening and require immediate medical intervention with urgent hospitalization. AHF is the main reason for hospitalization of patients who are ≥ 65 years old, of which 80–85% present with acute exacerbations of chronic HF, that is, acute decompensated heart failure (ADHF). AHF has a poor prognosis, with in-hospital mortality ranging from 4 to 7%, and a 60- to 90-day hospital readmission rate of approximately 30% [1].

Studies have shown that fluid retention is the main reason for hospitalization of ADHF patients [2, 3]. At present, a reduction in the degree of fluid retention is considered to be a priority treatment for ADHF [4, 5]. Diuretics have long been used as a cornerstone of the treatment of ADHF [6, 7]. Despite the extensive use of diuretics, the prognosis for inpatients with HF is not encouraging: 2–22% die during acute hospitalization [8, 9], 44% are readmitted within 6 months [10], and 33% die within 1 year [11]. It has been reported that long-term diuretic treatment might lead to activation of the neuroendocrine system in the kidney, electrolyte waste, renal insufficiency and progression of ADHF [12]. In addition, it is estimated that more than 20% of patients did not improve their symptoms after diuretic therapy, and furthermore diuretic resistance occurred in more than 30% of patients [13, 14].

The current treatment challenges of ADHF inspire alternative strategies, such as ultrafiltration. Ultrafiltration is a process of passing water and small to medium weight solutes through a semi-permeable membrane to reduce volume overload [15]. Ultrafiltration is recommended for patients with obvious volume overload to alleviate congestive symptoms and fluid weight (Class IIb, Level of Evidence: B) according to the 2016 European Society of Cardiology (ESC) Guidelines for the diagnosis and treatment of acute and chronic heart failure [6]. Studies have shown that ultrafiltration is a safe and effective way to remove excess fluid from HF patients [16–18]. Furthermore, a meta-analysis of 9 randomized controlled trials showed that compared with the diuretics group, ADHF patients in the ultrafiltration group had < 90-day readmission for HF, and a tendency to reduce cumulative hospitalization readmission [19]. In contrast, the concerns of using ultrafiltration are that ultrafiltration is associated with decreased renal function, mainly manifested by elevated serum urea and creatinine levels and increased risk of serious adverse events including renal failure, bleeding, and ultrafiltration catheter-related complications [20, 21]. Notably, the CARRESS-HF study [22] found that HF patients with worsening renal function had poor tolerance to blood ultrafiltration. Therefore, early use of ultrafiltration may be more effective than later use as a salvage therapy.

Early ultrafiltration may be an effective treatment for HF patients with volume overload. Both the RAPID-CHF trial [16] and the UNLOAD study [23] have demonstrated that early ultrafiltration (within 24 h of admission) is superior to diuretics in terms of weight loss and fluid removal. A number of clinical trials in other countries also provided evidence that early ultrafiltration can increase the sensitivity to diuretics of HF patients [24–26]. To date, there has been a paucity of studies on the effects of early ultrafiltration on volume management of Chinese patients with ADHF. In the present study, we compared the efficacy and safety of early ultrafiltration and conventional diuretic therapy on ADHF patients with volume overload. In the current situation of domestic low-flow ultrafiltration utilization in clinical practice, this study might provide evidence for clinicians to apply early ultrafiltration in the treatment of hypervolemic ADHF patients.

## Methods

Our study adheres to CONSORT guidelines and the intervention is described in accordance with the CONSORT checklist. This was a single center, prospective, randomized, controlled trial on the efficacy of ultrafiltration versus intensive diuretic treatment for patients with ADHF and hypervolemia. Patients were enrolled from April 2018 to September 2019 in the cardiac intensive care unit of our hospital. The ethics committee approved the study (Approval number: 2009-226) and all patients signed informed consent before participating.

### Patients

#### Inclusion criteria

Patients were ≥ 18 years old and were randomized after clinical evaluation. All patients met the diagnostic criteria for ADHF with volume overload. The diagnosis of ADHF was based on the 2017 ACC/AHA/HFSA guidelines for the management of heart failure [27]. A patient was judged to be hypervolemic according to at least 1 of the following criteria: (1) Moist rales in the lungs; (2) X-ray chest radiograph showing pulmonary congestion, pulmonary edema, or pleural effusion; (3) Congestive hepatomegaly or ascites; (4) Jugular venous pulse > 10 cm; (5) Lower limb edema (6) B-type natriuretic peptide (BNP) > 400 pg/mL.

#### Exclusion criteria

(1) Systolic blood pressure ≤ 90 mmHg and poor peripheral circulation; (2) Contraindications to heparin anticoagulation; (3) Severe mitral or aortic valve stenosis; (4) Tricuspid valvular disease; (5) Acute right ventricular myocardial infarction; (6) Required dialysis or hemofiltration; (7) Life-threatening organ dysfunction caused by a dysregulated host response to infection [28].

### Study procedures

**Fig. 1:**
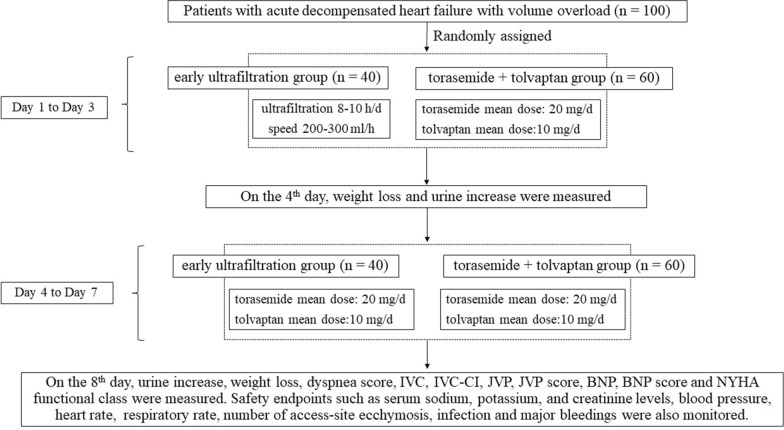
Flowchart of the study. Note: IVC: inferior vena cava; IVC-CI: collapse index of inferior vena cava; JVP: jugular venous pulse; BNP: B-type natriuretic peptide level; NYHA: New York Heart Association

Within 24 h of admission, patients were randomly assigned into an early ultrafiltration group or a torasemide + tolvaptan group. After 3 days of treatment, weight loss and the increase in urine output of the 2 groups were measured on the 4th day. From the 4th to the 7th day, patients in the early ultrafiltration group received sequential therapy of torasemide (mean dose: 20 mg/d) and tolvaptan (mean dose 10 mg/d). On the 8th day, the urine increase, weight loss, dyspnea score, inferior vena cava (IVC), inferior vena cava collapse index (IVC-CI), jugular venous pulse (JVP), JVP score, BNP and NYHA (New York Heart Association) functional class of the 2 groups were measured. Safety endpoints including respiratory and heart rates, blood pressure, serum concentrations of sodium, potassium and creatinine, number of access-site ecchymosis and infections, and major bleedings were also monitored. The flowchart of the study is shown in Fig. 1.

Patients randomized to the early ultrafiltration group were treated with bedside ultrafiltration hemodialysis within 24 h of admission using the FQ-16 type HF ultrafiltration dehydration device (Beijing Hartcare Medical Technology Co., Ltd). The blood ultrafilter was Hemocor HPH400 (Mini Teach, US). The deep vein (femoral vein, internal jugular vein or subclavian vein) was punctured and an 8F double lumen inserted, or a puncture of the bilateral median cubital vein or jugular vein with 18G intravenous was made with an dwelling needle for establishing the extracorporeal circulation blood route. The extracorporeal circuit was installed according to the equipment instructions. Heparinized normal saline was used fully to pre-flush the pipeline and filter, and the pre-flushing volume was not less than 300 mL. After the bubbles in the pipeline and filter were fully discharged, the patient was connected. The blood pump speed was set to 25–40 mL/min, and the initial ultrafiltration speed was set to 200–300 mL/h (or determined by the responsible physician). During the ultrafiltration treatment, blood pressure and heart rate were recorded every 30 min. If blood pressure dropped, the ultrafiltration speed was reduced. Anticoagulation was performed using the standard heparin regimen. During the treatment, the activated partial thrombin time (APTT) was maintained at 65–85 s, and the heparin dose was adjusted according to APTT. The mean ultrafiltration time was 10.8 h/d and the responsible physician could add or subtract according to the condition of the patient. Oxygen inhalation, infection control and other routine medical treatments were applied to help correct HF.

Patients randomized to the torasemide + tolvaptan group were treated with intravenous loop diuretics (mean torasemide dose: 20 mg/d) and a vasopressin V2 receptor antagonist (mean torvaptan dose: 10 mg/d) within 24 h of admission, along with oxygen inhalation, local infection control (such as lungs) and other routine medical treatments to help correct HF.

### Study outcomes and assessment

*Primary efficacy endpoints*: changes in body weight and daily urine volume in the early ultrafiltration and torasemide + tolvaptan groups on the 4th and 8th day of treatment.

*Secondary efficacy endpoints*: changes in the dyspnea score, IVC diameter, IVC-CI, JVP and BNP in both groups on the 8th day of treatment; readmission and mortality rates at the 1 and 3 months follow-ups.

*Safety endpoints*: heart rate, respiratory rate, blood pressure, serum sodium, potassium, creatinine levels and major bleedings of patients in the early ultrafiltration and torasemide plus tolvaptan groups.

Weight was measured in kilograms and urine volume was measured in milliliter at randomization on the 4th day and 8th day after treatment. Weight loss was calculated as the difference between the randomization weight and that recorded on day 4 or day 8. Urine increase was the difference between the daily urine volume at randomization and the daily urine volume recorded on day 4 and day 8. The urine increase in the early ultrafiltration group did not include the amount of water removal by ultrafiltration. We used the dyspnea scoring criteria in the "Recommendations for Standardized Assessment of Dyspnea in Acute Heart Failure Syndrome" published by the European Heart Association's International Working Group on Acute Heart Failure" [29] to evaluate improvements of dyspnea in patients with HF.

*After treatment significant effects*: those patients with a dyspnea score increased by > 8 points;

*Valid*: those patients with dyspnea scores increased by > 4 points but < 8 points; *Ineffective*: those patients with a dyspnea score that improved by < 2 points). IVC diameter and IVC-CI were measured using vascular ultrasound. We graded the JVP and BNP scores according to the “European grading congestion in acute heart failure” [3]. B-type natriuretic peptide concentrations were measured using the Triage BNP Test (Biosite Inc., San Diego, US) [30].

#### Sample size

Based on the result of several published studies, after treatment the mean ± standard deviation of weight changes in ultrafiltration patients was − 2.0 ± 2.5, while that of the controls was − 0.6 ± 1.5. A sample size of 35 and 53 subjects in the ultrafiltration and control group (the sample size ratio of the 2 groups was 2:3) was found to be sufficient to reveal this difference with 80% power at a 0.05 significance level. Considering a 10% dropout rate, a sample size of 40 and 60 patients was required for the 2 groups, respectively.

### Statistical analysis

Data were analyzed using SPSS ver. 17.0. Normally distributed continuous variables are presented as the mean ± SD and comparisons made between normalized data sets employed an unpaired *t*-test. Categorical variables are reported as frequencies and percentages and potential significance differences assessed using chi-squared or Fisher’s exact tests. A difference was considered to be statistically significant when the *P *value was < 0.05 (both sides).

## Results

### Patient baseline characteristics

The mean age of the cohort of patients was 72.35 ± 10.13 years, with males accounting for 55% of them. Of the patients, circa 61% had prior HF. The mean JVP was 15.72 ± 3.71 cm. With regard to the medication the patients were receiving before hospitalization, 99% were on β-adrenoceptor antagonists and ACEI/ARB, and 100% on diuretics. The mean serum creatinine concentration of the study population was 139.26 ± 79.37 μmol/L. The demographic and clinical characteristics of the two groups were comparable (Table 1).

**Table 1:** Baseline demographics and clinical characteristics of the patients with ADHF, according to the treatment received (n = 100)

	Early ultrafiltration group (n = 40)	Torasemide plus tolvaptan group (n = 60)	*P* value
Age (years)	70.6 ± 10.44	73.52 ± 9.83	0.159
Male gender (%)	22 (55.0)	33 (55.0)	1.000
Weight (kg)	69.45 ± 12.12	65.63 ± 8.541	0.067
Prior heart failure (%)	23 (57.5)	38 (63.3)	0.558
JVP (cm)	15.00 ± 2.63	16.20 ± 4.24	0.084
Comorbidity			
Coronary artery disease	28 (70.0)	43 (71.7)	0.857
History of hypertension (%)	32 (80.0)	48 (80.0)	1.000
Diabetes (%)	26 (65.0)	38 (63.3)	1.000
Dilated cardiomyopathy	9 (22.5)	8 (13.3)	0.232
Arrhythmia	21 (52.5)	24 (40.0)	0.218
Pulmonary infection	14 (35.0)	24 (40.0)	0.614
Post-PCI	10 (25.0)	10 (16.7)	0.307
Renal dysfunction	13 (32.5)	16 (26.7)	0.529
Hepatic dysfunction	3 (7.5)	1 (1.7)	0.299
Laboratory measurements			
Serum sodium (mmol/L)	140.41 ± 4.58	141.37 ± 3.85	0.281
Serum potassium (mmol/L)	4.03 ± 0.72	3.83 ± 0.63	0.156
Serum creatinine (μmol/L)	155.70 ± 72.05	128.30 ± 82.66	0.091
BNP (pg/mL)	1266.21 ± 1082.59	1310.50 ± 982.35	0.839
Medications			
β-adrenoceptor antagonists	40 (100.0)	59 (98.3)	1.000
ACEI/ARB	40 (100.0)	59 (98.3)	1.000
Diuretic	40 (100.0)	60 (100.0)	1.000
Positive inotropic agents	27 (67.5)	40 (66.7)	0.931
Antithrombotic drugs	25 (62.5)	45 (75.0)	0.181
Lipid-lowering drugs	30 (75.0)	35 (58.3)	0.087

*JVP* jugular venous pulse, *PCI* percutaneous coronary intervention, *BNP* B-type natriuretic peptide

### Primary efficacy endpoint

**Fig. 2:**
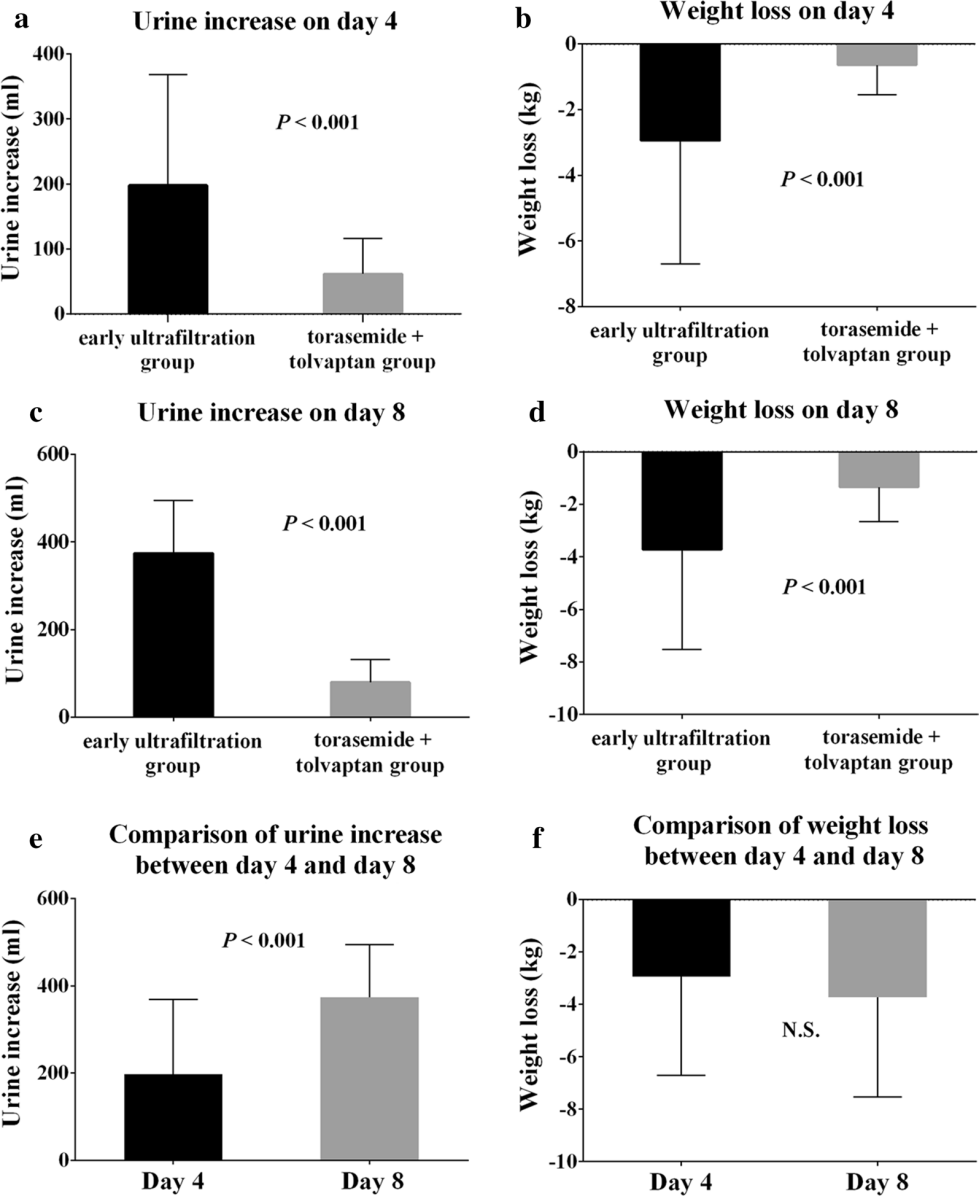
Urine increase (**a**, **c**) and weight loss (**b**, **d**) of patients on day 4 and day 8 according to the treatment received (n = 100). Comparison of **e** urine increase and **f** weight loss of patients in the early ultrafiltration group on days 4 and 8

The use of early ultrafiltration is associated with better control of volume. Three days after treatment, the early ultrafiltration group had a significantly greater weight loss than the torasemide + tolvaptan group (− 2.94 ± 3.76 kg vs − 0.64 ± 0.91 kg, *P* < 0.001). The mean urine increase on day 4 after treatment was greater in the early ultrafiltration group compared with the torasemide + tolvaptan group (198.00 ± 170.70 mL vs 61.77 ± 54.67 mL, *P* < 0.001). From day 4 to day 7, patients in the early ultrafiltration group received sequential therapy of torasemide and tolvaptan. On the 8th day, the early ultrafiltration group had a significantly greater weight loss than the torasemide plus tolvaptan group (− 3.72 ± 3.81 kg vs − 1.34 ± 1.32 kg, *P* < 0.001). The mean urine increase on day 8 after treatment was greater in the early ultrafiltration group than in the torasemide plus tolvaptan group (373.80 ± 120.90 mL vs 79.5 ± 52.35 mL, *P* < 0.001). Furthermore, we found that the mean urine increase of patients in the early ultrafiltration group on day 8 was significantly greater than that of patients in the early ultrafiltration group on day 4 (*P* < 0.001). However, no differences in weight loss were measured in patients who received early ultrafiltration with and without torasemide plus tolvaptan (Fig. 2).

### Secondary efficacy endpoint

**Fig. 3:**
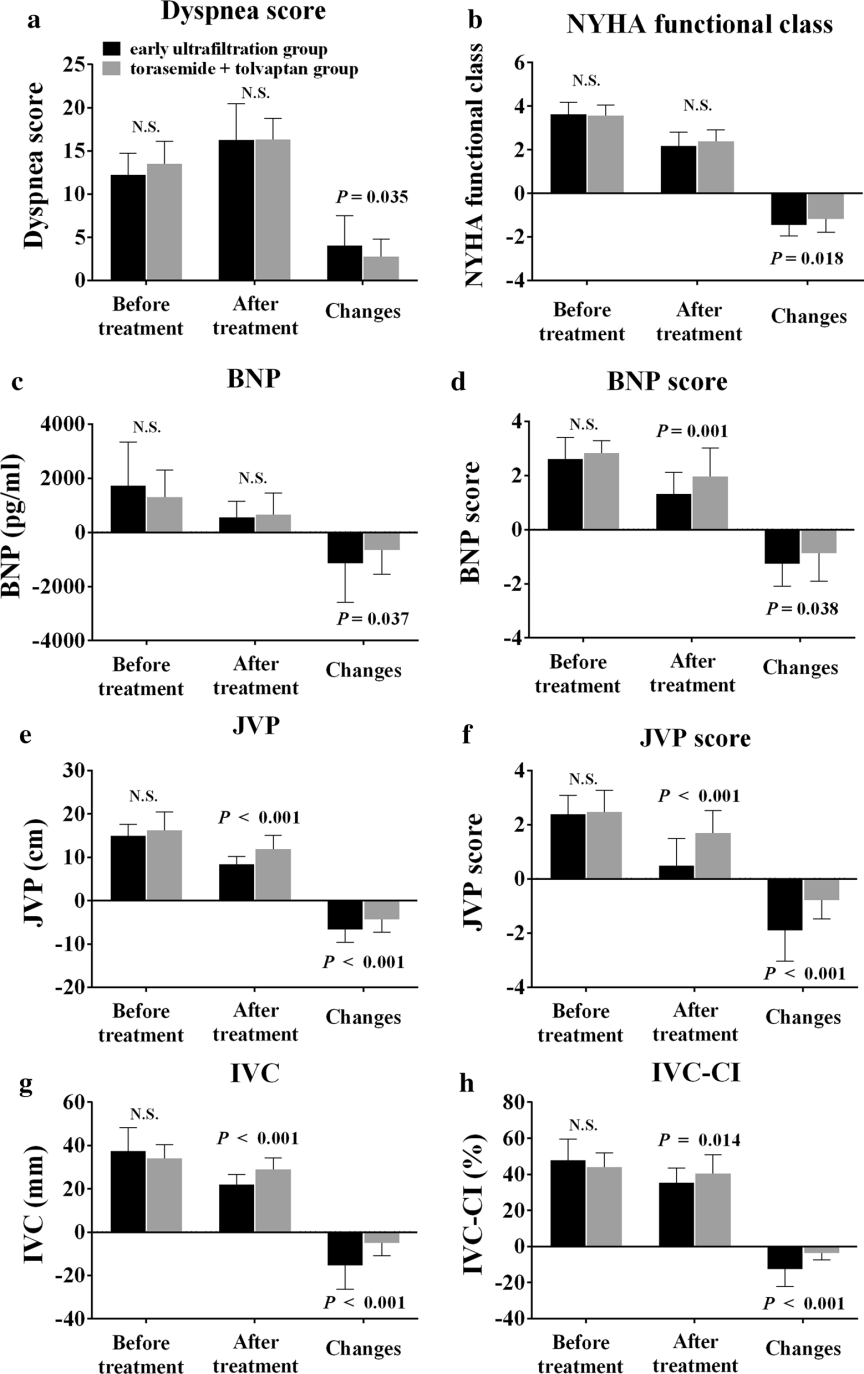
Comparison of **a** dyspnea scores, **b** NYHA functional class, **c** BNP, **d** BNP scores, **e** JVP, **f** JVP scores, **g** IVC, **h** IVC-CI between early ultrafiltration group (n = 40) and torasemide plus tolvaptan group (n = 60). Note: NYHA: New York Heart Association; JVP: jugular venous pulse; BNP: B-type natriuretic peptide concentration; IVC: inferior vena cava; IVC-CI: collapse index of the inferior vena cava. n.s. = there was no significant difference between the 2 groups

The better control of volume was also reflected in the greater reduction of JVP (− 6.65 ± 2.99 vs − 4.30 ± 2.98, *P* < 0.001), JVP score (− 1.9 ± 1.13 vs − 0.78 ± 0.69, *P* < 0.001), IVC diameter (− 15.35 ± 11.03 vs − 4.98 ± 6.00, *P* < 0.001) and IVC-CI (− 12.43 ± 9.87 vs − 3.50 ± 3.89, *P* < 0.001) in the early ultrafiltration group compared to the torasemide plus tolvaptan group on day 8. The advantage of early ultrafiltration in the treatment efficacy of ADHF lies in the significant increase in the dyspnea score (4.08 ± 3.44 vs 2.77 ± 2.03, *P* = 0.035) and the greater reduction in BNP (− 1144 ± 1435 vs − 654.02 ± 889.65, *P* = 0.037) and the BNP score (− 1.27 ± 0.816 vs − 0.87 ± 1.03, *P* = 0.038) (Fig. 3).

We also measured the readmission and mortality rates in the 2 patient groups at the 1-month and 3-month follow-ups and found no significant differences between the 2 groups (Table 2). However, the 3-month readmission rate data revealed a lower trend in the early ultrafiltration group. In a future study, if the follow-up time is longer or the sample size is further expanded, a significant difference may well be found.

**Table 2:** Comparison of readmission rates and mortality according to the treatment received (n = 100)

	Early ultrafiltration group (n = 40)	Torasemide plus tolvaptan group (n = 60)	*P*-value
1-month follow-up			
Readmission rates (%)	7 (17.5)	12 (20.0)	0.755
Mortality (%)	0 (0.0)	1 (1.7)	1.000
3-months follow-up			
Readmission rates (%)	8 (20.0)	22 (36.7)	0.075
Mortality (%)	0 (0.0)	1 (1.7)	1.000

### Safety endpoints

Of the 40 patients in the ultrafiltration group, 2 patients had subcutaneous congestion at the puncture site and no patient had an infection or major bleeding. We also compared the heart rate, blood pressure, respiratory rate, serum sodium, potassium and creatinine concentrations of patients who received ultrafiltration before and after treatment, and found no significant clinical change was detected (Table 3). There was no significant difference in the above indicators between the early ultrafiltration and torasemide plus tolvaptan groups.

**Table 3:** Comparison of safety endpoints according to the treatment received (n = 100)

Safety endpoint	Early ultrafiltration group (n = 40)		Torasemide plus tolvaptan group (n = 60)	
	Before treatment	After treatment	Before treatment	After treatment
Heart rate (beats/min)	85.25 ± 18.37	84.20 ± 14.32	82.02 ± 14.65	82.72 ± 15.78
Systolic blood pressure (mmHg)	124.98 ± 19.14	128.40 ± 19.55	128.40 ± 19.55	129.12 ± 18.69
Diastolic blood pressure (mmHg)	68.63 ± 11.04	67.38 ± 10.07	71.93 ± 11.28	68.62 ± 10.38
Respiratory rate (beats/min)	20.28 ± 2.89	21.68 ± 3.53	19.67 ± 2.61	20.22 ± 3.29
Serum sodium (mmol/L)	137.12 ± 22.20	142.23 ± 4.66	141.37 ± 3.85	142.50 ± 4.06
Serum potassium (mmol/L)	4.03 ± 0.72	4.06 ± 0.54	3.83 ± 0.63	3.95 ± 0.55
Serum creatinine (μmol/L)	155.70 ± 72.05	154.27 ± 88.46	128.30 ± 82.66	126.57 ± 75.92
Access-site ecchymosis (N)	–	2	–	–
Access-site infection (N)	–	0	–	–
Major bleedings (N)	–	0	–	–

Data values are presented as the mean ± SD. For all comparisons between the two treatments, *P* was n.s

## Discussion

Excessive water storage due to a sodium ion imbalance is the main reason for hospitalization of ADHF patients, with the main clinical manifestations being systemic and pulmonary congestion. Relieving congestion is an important treatment for ADHF. In the present study, we found that early ultrafiltration resulted in more weight loss and a greater urine output in hypervolemic ADHF patients compared to the torasemide plus tolvaptan treated group. Furthermore, when patients in the early ultrafiltration group received sequential therapy of torasemide and tolvaptan, the effect of reducing the volume load was also more significant than that of intensive diuretics therapy. In addition, early ultrafiltration was applied safely during the study period.

Consistent with previous studies [17, 31, 32], our data from the first 3 days of treatment showed that ultrafiltration alone significantly improved congestion by removing fluid and reduced more of the volume load than a diuretic infusion alone. On the 8th day, the urine increase and weight loss of patients who received early ultrafiltration and sequential toracemide or tolvaptan was significantly higher than for patients treated with toracemide plus tolvaptan. In addition, patients who were given early ultrafiltration and sequential therapy with diuretics had a greater urine output increase than those who received ultrafiltration alone. These findings all indicated that ultrafiltration increased the sensitivity of patients to diuretics. It is well known that the long-term use of diuretics can impair the ability of the kidneys to produce urine with a particular dose of diuretic. Despite increasing the diuretic dose, fluid retention and congestion symptoms were not adequately controlled [33]. After removing a large quantity of isotonic fluid, ultrafiltration relieved the symptoms of congestion, improved exercise capacity and the heart filling pressure, and restored the diuretic response in patients who had been diuretic resistance [34]. Multiple observations suggested that early ultrafiltration could reduce diuretic exposure and improve patient responsiveness to these drugs [17, 32, 35, 36].

Although our sample size already had a 95% test of power, there was no significant difference in weight loss in the early ultrafiltration group between day 4 and day 8. There was a linear relationship between weight loss and urine increase (correlation coefficient − 0.254, regression coefficient − 0.005, *P* = 0.011, n = 100). Therefore, it is possible that these patients may have a bias in weight change caused by constipation or due to a large amount of short-term eating, but this speculation can only be confirmed in future studies with a larger cohort of patients.

The dyspnea scores of the 2 groups exhibited different degrees of improvement after treatment, but the difference in scores before and after treatment in the early ultrafiltration group was > 4 points, which meant that the efficacy of treatment was significantly greater than that of the intensive diuretic group. Ultrafiltration relieves dyspnea by reducing pulmonary vein pressure and pulmonary congestion through mechanical dehydration, and our results are consistent with the results of the RAPID-CHF trail [16].

Furthermore, BNP was also observed in the 2 groups. As a cardiac neurohormonal secreted from the ventricle, BNP reflects ventricular volume expansion and pressure overload [37, 38]. Elevated BNP is related to the NYHA classification and prognosis [39, 40]. Early ultrafiltration and sequential therapy with diuretics reduces neurohormonal activation, which can be demonstrated by a decrease in the plasma BNP concentration and BNP scores, with the reduction being significantly greater than in the intensive diuretic group. Ultrafiltration can improve diuresis by reversing the braking phenomenon through “diuretic holidays” and reducing the activation of neurohormones [32, 33].

In addition, changes in JVP, the JVP score, NYHA functional class, IVC diameter and IVC-CI values before and after treatment in the early ultrafiltration group were significantly greater than in the intensive diuretic group, indicating that ultrafiltration therapy had significant effects in correcting the clinical symptoms of sodium concentrations and therefore water retention in ADHF and in improving volume responsiveness. Ultrafiltration removes water from the blood through the dialysis membrane by a convective transport mechanism, which reduces the effective blood volume and improves the systemic circulation [41, 42].

We also observed 1-month and 3-month mortality at the follow-ups and there was no difference between the 2 groups. In the early ultrafiltration group, the readmission rate for ADHF during the 3-month follow-up suggested a trend lower than that of the intensive diuretic group, although statistical significance was not reached. Improved clinical outcomes and use of ultrafiltration may mean less utilization of resources for ADHF [31].

Since the goal is to remove excess fluid inside and outside blood vessels without further activating the neuroendocrine system and/or deteriorating renal function [32], the safety of ultrafiltration is also a concern. From the safety end point, ultrafiltration plus tolvaptan treatment did not significantly affect blood pressure, heart or respiratory rates, blood creatinine, sodium or potassium ion concentrations in patients with ADHFC, which is equivalent to intensive diuretic therapy and had good safety. Early ultrafiltration reduces edema through mechanical isotonic dehydration while maintaining blood pressure and heart rate stability. The concentrations of creatinine, sodium and potassium ions will not be diminished by dehydration, and the reduced blood volume is stabilized by the intra-osmotic supplementation of interstitial edema, keeping the above indicators stable, which is consistent with previously reported findings [43].

Interestingly, 61% of our ADHF patients had prior HF with 99% on beta-blockers and ACEI/ARB and 100% on diuretics, probably because 71% had coronary artery disease, 80% hypertension and 64% diabetes. These comorbidities may give rise to the extensive use of beta-blockers, ACEI/ARB and diuretics for therapy.

In the present study, the control group were administered tolvaptan in addition to a loop diuretic (torasemide) at regular doses. However, tolvaptan is not a routine first-line drug treatment for ADHF in the European Heart Failure guidelines, but it is a routine first-line drug for ADHF in Japan. The EVEREST, TACTICS-HF and other previous studies found that the early use of tolvaptan in patients with ADHF could significantly reduce body weight, increase the negative balance of fluid, relieve congestion symptoms, without obvious short-term and long-term adverse reactions [44–46]. The Chinese expert recommendations on volume control in heart failure management published in Chin J Heart Fail & Cardiomyopathy (2018) also recommended early use of tolvaptan in ADHF patients [47]. It is worth noting that since we mainly wanted to observe the efficacy and safety of early ultrafiltration, torasemide (20 mg/d) and tolvaptan (10 mg/d) used in our control group were routine doses according to published clinical guidelines [48].

One limitation of the present study was that patients were enrolled from a single center, so there was only a relatively small sample size. A large cohort study will be required to follow up cardiac events, rehospitalization rates, mortality and other indicators 3- and 6-months after discharge. In addition, the ejection fraction (%) was not measured because we mainly focused on short-term efficacy, which may limit the comparison of this study with previous and future ones. Finally, treatment cannot be blind, but it has little effect on weight loss and increases in urine output.

## Conclusions

In summary, our single center trial has demonstrated that early ultrafiltration effectively and safely reduces volume overload in patients with ADHF. A treatment strategy of early ultrafiltration and sequential therapy with diuretics may restore patients’ responsiveness to diuretics and help them achieve an easing of dyspnea symptoms and improve their cardiac function. Multicenter clinical trials and longer follow-ups are still needed to validate our results. Future studies should focus on understanding the effect of ultrafiltration on the prognosis of HF, identify the population most likely to benefit from ultrafiltration therapy, select the best indication of ultrafiltration therapy and explore the best timing for the initiation of ultrafiltration.

## Data Availability

The datasets used and/or analyzed during the current study are available from the corresponding author on reasonable request.

## Abbreviations

AHF: Acute heart failure
ADHF: Acute decompensated heart failure
APTT: Activated partial thrombin time
BNP: B-type natriuretic peptide
HF: Heart failure
IVC: Inferior vena cava
IVC-CI: Inferior vena cava collapse index
JVP: Jugular venous pulse
NYHA: New York Heart Association
